# Therapeutic effects of simvastatin on Galectin-3 and oxidative stress parameters in endotoxemic lung tissue

**DOI:** 10.1042/BSR20180308

**Published:** 2018-06-27

**Authors:** Hatice Yorulmaz, Elif Ozkok, Engin Kaptan, Gulten Ates, Sule Tamer

**Affiliations:** 1 School of Nursing, Halic University, Istanbul, Turkey; 2 Department of Neuroscience, Aziz Sancar Institute of Experimental Medicine, Istanbul University, Istanbul, Turkey; 3 Department of Biology, Faculty of Sciences, Istanbul University, Istanbul, Turkey; 4 Department of Physiology, Istanbul Medical Faculty, Istanbul University, Istanbul, Turkey; 5 Department of Physiology, Faculty of Medicine, Istanbul Yeni Yuzyil University, Istanbul, Turkey

**Keywords:** Antioxidant enzymes, galectins, lipopolysaccharides, oxidative stress, simvastatin, TBARS

## Abstract

Galectins constitute of a soluble mammalian β-galactoside binding lectin family, which play homeostatic roles in the regulation of the cell cycle, and apoptosis, in addition to their inflammatory conditions. Galectin-3 has an important role in the regulation of various inflammatory conditions including endotoxemia, and airway inflammation. Statins, the key precursor inhibitors of 3-hydroxyl-3-methyl coenzyme A (HMG-CoA) reductase, may prevent the progression of inflammation in sepsis after prior statin treatment. Endotoxemia leads to the formation of oxidative stress parameters in proteins, carbohydrates, and DNA. In the present study, we aimed to show the effects of simvastatin on Galectin-3, and glutathione reductase (GR), glutathione peroxidase (GSH-Px), superoxide dismutase (SOD), and thiobarbituric acid reactive substances (TBARS) levels in lung tissue of rats which were treated with lipopolysaccharides (LPS) during the early phase of sepsis. Rats were divided into four groups as the control, LPS (20 mg/kg), simvastatin (20 mg/kg), and simvastatin+LPS group. Galectin-3 expression in formalin-fixed paraffin-embedded lung tissue sections was demonstrated by using the immunohistochemistry methods. There were reduced densities, and the decreased number of Galectin-3 immunoreactivities in the simvastatin+LPS group compared with the LPS group in the pneumocytes, and in the bronchial epithelium of lung tissue. In the LPS group, GR, GSH-Px, and SOD were found lower than the levels in simvastatin-treated LPS group (*P*<0.05, *P*<0.01, *P*<0.01 respectively) in the lung tissue. However, TBARS decreased in the simvastatin+LPS group compared with the levels in LPS group (*P*<0.001). Simvastatin attenuates LPS-induced oxidative acute lung inflammation, oxidative stress, and suppresses LPS-induced Galectin-3 expression in the lung tissue.

## Introduction

Sepsis is defined as the development of multiple organ failure, and inflammatory responses including cellular, and metabolic changes against bacterial invasion in the host tissue [1]. The lung is mostly the first affected organ in septic shock. A membrane component of Gram-negative bacteria lipopolysaccharide (LPS), which is used in experimental acute lung injury (ALI) models, and interacts with toll-like receptor 4 (TLR4), which initiates an inflammatory, and immune response. TLR4 mediates the host response to LPS by promoting the activation of tumor necrosis factor-α (TNF-α), interleukin-1β (IL-1β), and IL-6 genes in inflammatory cells. However, the inflammatory cells produce cytotoxic molecules, and free radical-damaged lung tissue causes ALI [2].

The reactive oxygen species (ROS) causes tissue injury in the instances of imbalances between the ROS, and antioxidants defense mechanisms [3].

Superoxide radical, is one of the important radicals, which may be dismutated to hydrogen peroxide (H_2_O_2_) by Superoxide dismutase, and then H_2_O_2_ reduced to H_2_O in the presence of reduced form of Glutathione (GSH) by Glutathione peroxidase (GSH-Px) enzyme, also formed oxidized form of Glutathione (GSSG). Glutathione is known as the most abundant intracellular antioxidant molecule, which is reacted with ROS and superoxide radical, and converted into the oxidized form glutathione (GSSG). Glutathione reductase (GR) is catalyzed from oxidized form GSSG to reduced form GSH in the presence of NADPH [4].

The ROS and oxidative stress have important roles in the development of sepsis and increase the pulmonary inflammation [5].

Thiobarbituric acid reactive substances (TBARS) is used to measure the lipid peroxidation products, which show oxidative stress in the cells and tissues; sepsis pathophysiology is accompanied by an increase in serum TBARS [6].

Galectins, β-galactoside-binding animal lectins, are produced by the inflammatory cells, and have homeostatic roles in various pathologic conditions [7]. Current research indicated that Galectin-3 contributes to diverse physiologic, and pathologic processes including endotoxemia, and airway inflammation through a multitude of complex signaling pathways [8]. Galectin-3 was demostrated as a biomarker in many inflammatory diseases which involved increased free radical production [9]. Researchers showed that exogenously given Galectin-3 was to have an effect on oxidative stress by increasing the superoxide radical production from human neutrophils [10,11].

LPS/Galectin-3 interaction provides oligomerization of Galectin-3, which stabilizes LPS monomers by increasing LPS aggregates, and causing the activation of neutrophils in inflammatory conditions [12].

Simvastatin was shown to exhibit important anti-inflammatory and antioxidant effects [13]. The use of statins in sepsis is occured from some experimental and subsequent clinical studies, which showed that simvastatin reduces cytokine levels and leukocyte counts in acute respiratory distress syndrome, ALI, or sepsis [14]. Researchers in recent studies reported the preventive roles of statin pretreatment on both experimental and clinical sepsis models [15].

It was observed increased expression of Galectin-3 when treated with simvastatin in prion infection [16].

However, the number of studies associated with the effect of simvastatin on Galectin-3 in LPS-treated rats is scarce in the literature. In the present study, we aimed to show the effects of simvastatin on the oxidative parameters: GR, GSH-Px, superoxide dismutase (SOD) antioxidants, TBARS, and Galectin-3 in lung tissue in endotoxemia.

## Materials and methods

### Experimental groups

The study was approved from the Animal Experiments Local Ethics Committee of Istanbul University (Resolution No: 2012/138). A sample of 32 male adult Wistar albino rats weighing 200–250g was used in the experiments. Rats were randomly divided into four groups as the control group, LPS group, simvastatin group, and LPS+simvastatin group

### Experimental procedures

The animals were fed with a commercial diet and tap water *ad libitum*, were housed in cages and kept at a controlled temperature (22 ± 2°C) and humidity (55–60%) with a 12-h light/dark cycle.

The intraperitoneal sepsis model was created 1 ml of LPS (dissolved in 0.9% NaCl) from *Escherichia coli* O127:B8 (Sigma Aldrich, Product No: L5668) using a dose of 20 mg/kg. Oral simvastatin (20 mg/kg) (Sigma Aldrich, Product No:S0650000, dissolved in 0.9% NaCl) was administered through oral gavage for 1 ml/5 days [13]. In the simvastatin+LPS group, LPS was injected after 5 doses of simvastatin administration. Rats were decapited after 4 h in the LPS and simvastatin+LPS groups.

### Histological procedures

Five-micrometer-thick liver sections were placed on polylysine-coated slides, and were stained using hematoxylin and eosin (H&E). The slides were evaluated under a light microscope. The lung sections were evaluated for the degree of inflammatory cell infiltration; the scoring criteria were as follows: grade 0: absent; grade 1: rare; grade 2 as moderate; and grade 3, as extensive. Photomicrographs were taken using an Olympus BX53F light microscope equipped with an Olympus DP27 digital camera.

### Immunohistochemical procedures

The lung sections (4–5-μm thick) mounted on poly-l-lysine-coated glass slides were deparaffinized and rehydrated. The sections were then heated with citrate buffer (0.1 M, pH 6) for 15 min in a microwave oven for antigen retrieval. To block endogen peroxidase, sections were incubated in 3% hydrogen peroxide for 10 min. After repeated washing with PBS, protein blocking was performed with the blocking solution of histostain plus kit component (Novex by Life Technologies) for 10 min. Polyclonal rabbit anti-Galectin-3 (sc-20157, 1:50, overnight) were applied as primary antibody, and broad spectrum second antibody (Novex by Life Technologies) were used. After flushing, the sections were treated with peroxidase-conjugated streptavidin (Novex by Life Technologies), and then aminoethyl carbazole chromogen were used for the visualization of the antibody reaction. The sections were counterstained using Mayer’s hematoxylin and examined under a light microscope. H-score: The score was obtained using the formula: [(3 × percentaged value of the strongly stained cells) + (2 × percentaged value of the moderately stained cells) + (the percentaged value of the weakly stained cells)], giving a range of 0–300.

### The identification of the enzyme-linked immunosorbent assay for GR, GSH-Px, and SOD

Lung tissue GR, GSHPx, and SOD levels were identified in the ELISA using biotin-based double antibody sandwich tehcnique at 450 nm (sensitivity levels as: 0.025 ng/l for GR; 2.21 U/ml for GSHPx; and 0.016 ng/ml for SOD).

### TBARS procedure

Lipid peroxide levels in the lung tissue were calculated by measuring the levels of TBARS. In this method, lung tissue homogenates were reacted with thiobarbituric acid (TBA, Sigma Aldrich) reagent which contained trichloroacetic acid (TCA, Sigma), concentrated hydrochloric acid (HCl, Merck), and butylated hydroxytoluene (Sigma), boiled for 15 min, and then cooled, and centrifuged. TBARS levels were measured in supernatant fraction using spectrophotometry (Shimadzu, Japan) at 532 nm, and the concentrations were calculated and expressed as nmol/g tissue [17].

### Statistical analysis

The Statistical package for the Social Sciences (SPPS) 17.0 statistical software (SPSS, Inc., Chicago, IL, U.S.A.) was used in the statistical analysis. The mean values were compared using Tukey’s multiple comparison test followed by one-way ANOVA. The data were presented as the mean ± standard deviation (SD). *P*<0.05 was considered as statistically significant.

## Results

### Histological results

**Figure 1:**
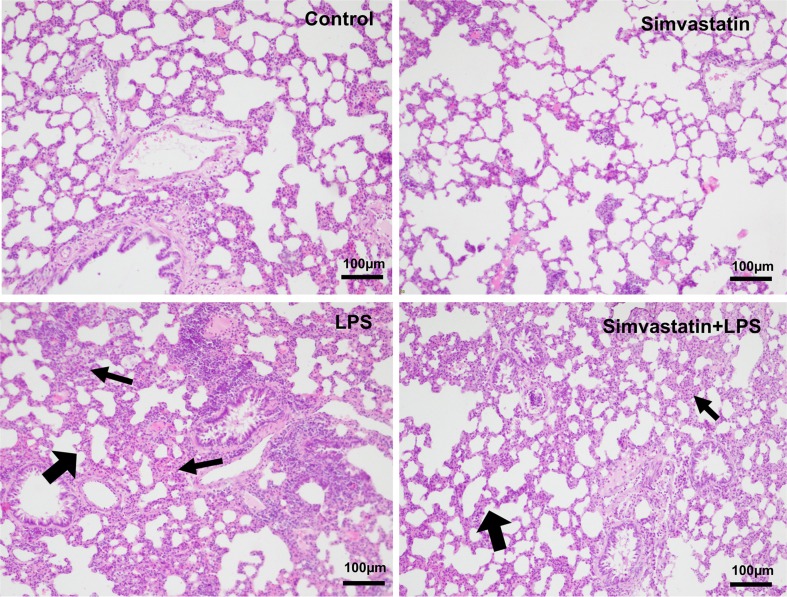
Section of the lung tissue from the groups stained with H&E

**Figure 2:**
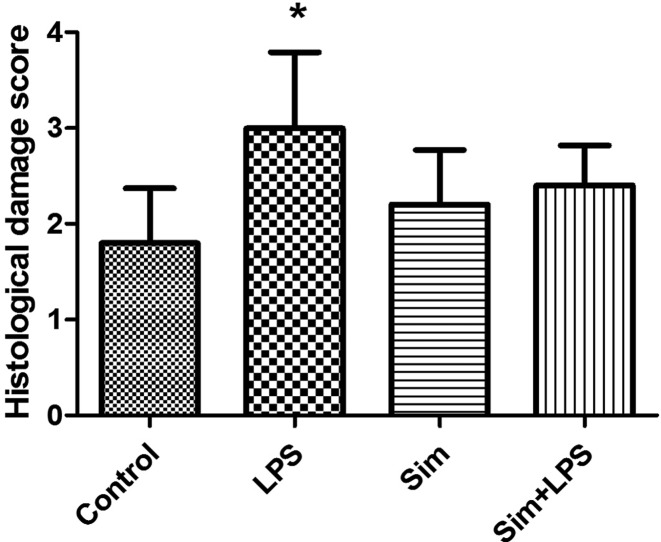
The histological damage score of the groups, ^*^P≤0.01 vs. control group

The control and simvastatin groups were found to have normal histological structure in the H&E staining. Intensive inflammation was detected in lung sections in the LPS group. The LPS group was observed to have higher histological score damage than the other groups (*P*<0.01). Though insignificant, the lung damage was observed to reduce in simvastatin+LPS group (Figures 1 and 2)

### Immunohistochemical results

**Figure 3:**
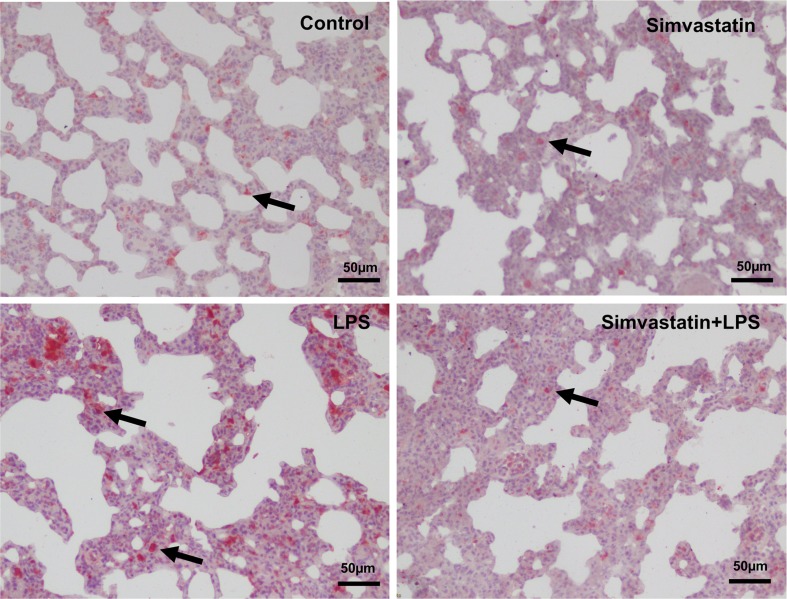
Galectin-3 immunoreactivity in the experimental groups

**Figure 4:**
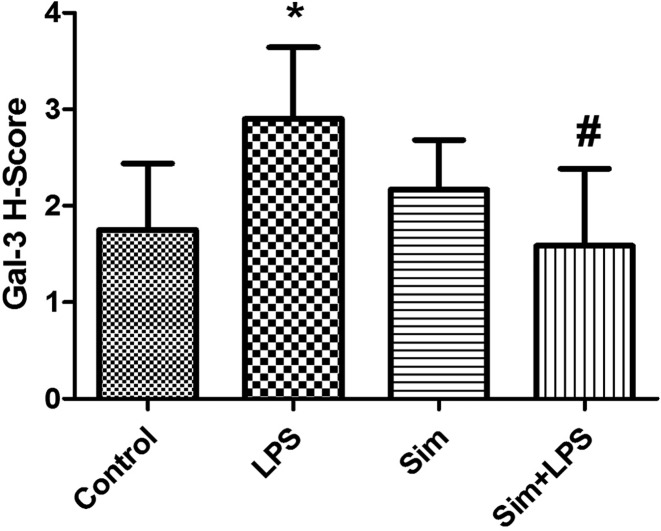
Galectin-3 immunoreactivity score in the experimental groups. ^*^P≤0.05 compared with control group, ^#^*P*≤0.05 compared with LPS group.

Reduced densities and decreased number of Galectin-3 immunoreactivities in the simvastatin+LPS group were shown compared with the LPS group in pneumocytes and bronchial epithelium of lung tissue (*P*≤0.05) (Figures 3 and 4).

### The enzyme-linked immunosorbent assay results of GR, GSH-Px, and SOD

**Figure 5:**
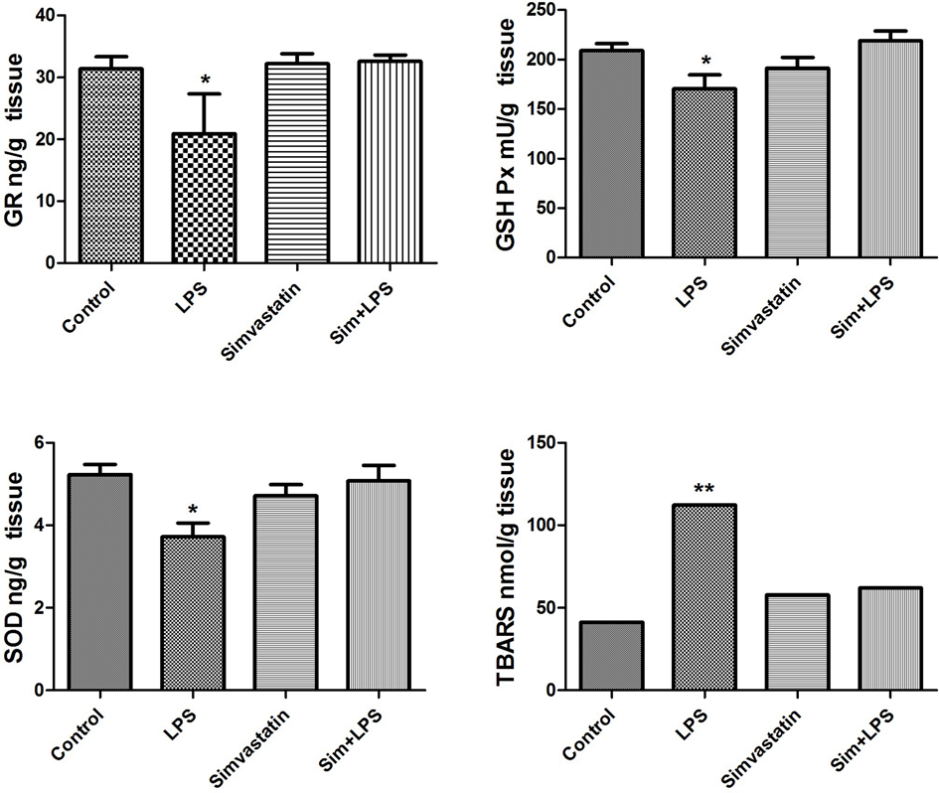
Oxidative stress parameters (GR, GSH-Px, SOD, and TBARS) and levels in the experimental groups (*P<0.05 and **P<0.01 )

GR was found statistically lower in the lung tissue in LPS compared with the control and simvastatin groups (*P*<0.05, for both). GR was found higher in simvastatin-treated LPS group compared with LPS group (*P*<0.05) (Figure 5).

Lung tissue GSH-Px levels significantly decreased in LPS group compared with the control and simvastatin+LPS groups (*P*<0.05 and *P*<0.01 respectively) (Figure 5).

In the LPS group, lung tissue SOD levels were found to be lower compared with the control and simvastatin+LPS groups (*P*<0.01, for both) (Figure 5).

### TBARS result

TBARS levels significantly decreased in the simvastatin+LPS group compared with the LPS group (*P*<0.001) and there is no significant meaning in the other experimental groups compared with that of control (*P*>0.05) (Figure 5).

## Discussion

The most sensitive and critically infectious organ causing sepsis is the lung [18]. Sakaguchi et al. [19] demonstrated that LPS treatment caused increased peroxidation and membrane damage in experimental studies by reducing the antioxidant levels.

The imbalance of oxidant/antioxidant levels are known to cause tissue damage in endotoxemia and sepsis [20].

Researchers in a number of studies evidenced that elevated-free radicals in addition to decreased antioxidant capacity of pulmonary vascular tissue may effect the prognosis of patients diagnosed with ALI [21].

Because it is important to maintain cell survival and homeostasis in the organism, there are antioxidative defense molecules and enzymes such as GSH, SOD, GSH-Px, GR, and catalase (CAT) against the free radical attack [4]. GR is a critical enzyme in the regeneration of the GSSG to reduced form GSH in the cell. Both SOD and GSH-Px are consumed in the removal of the superoxide radicals generating H_2_O_2_ in septic conditions [22]. Compatible with the literature, we found decreased tissue levels among GR, GSH-Px, and SOD antioxidant enzymes in LPS-treated rats compared with the controls in our study [23]. In the simvastatin-treated LPS group, GR, GSH-Px, and SOD levels increased closer to the control’s results in the same way.

TBARS represented the content of malondialdehyde (MDA)—an end product of lipid peroxidation in the tissue [24]. In our study, TBARS levels significantly increased in rats with LPS. Statins showed antioxidant effect against the production of the free radicals [25].

In our study, simvastatin treatment improved TBARS levels. Simvastatin may inhibit oxidant formation by suppresing the effects of ROS, and antioxidant enzymes up-regulation, which blocks free radicals [26].

Most of the inflammatory cells are expressed Galectin-3 and its expression level can alter related to external or internal stimuli. Galectin-3 play a significant role in acute inflammatory response such as neutrophil activation and adhesion [27], chemoattraction of monocytes or macrophages, and opsonization of apoptotic neutrophils [28]. It was shown that alveolar macrophages secreted Galectin-3 when they were treated with *Streptococcus pneumoniae* membrane fraction [29]. Henderson and Sethi [30] found that macrophage activation through IL-4 and IL-13 induced expression and release of Galectin-3. It was also shown that human macrophages polarized into M1, M2a, and M2c subtypes when they were treated with granulocyte macrophage colony-stimulating factor (GM-CSF), IFN-γ/LPS. These subtypes exhibited an increase level of Galectin-3 expression in the cytosol [31]. In this present study, Galectin-3 expression in alveolar macrophages increased in LPS-treated animals. However, simvastatin injection decreased its expression in LPS-treated animals. Their immunoreactivity patterns looked like those of control. As a result, simvastatin improved the acute inflammatory changes occurred in the lungs of LPS-injected animals.

Researchers showed in a number of studies that Galectin-3 promoted vascular inflammation by inducing the expression of proinflammatory products in the macrophages [28].

The expression of Galectin-3 in inflammatory cells might lead to their enhanced survival, resulting with the exacerbated inflammation, in addition it might regulate inflammation through a variety of mechanisms [32]. A number of Galectin-3 immunoreactivities in the LPS group increased in pneumocytes and in the bronchial epithelium of lung tissue. Statin administration reduced plaque alectin-3 expression in addition to the plaque macrophage contents during the murine atherosclerosis [33]. Prior simvastatin administration decreased the densities, and the number of the Galectin-3 immunoreactivities in pneumocytes, and bronchial epithelium of lung tissue.

Sepsis is the major cause of ALI in clinical conditions. ALI is characterized by the intensive lung inflammation and alveolar damage, which may lead to multiorgan damage [34].

Extensive inflammatory cell infiltration was detected in LPS group in our study. Researchers in a retrospective study found that statin therapy was beneficial for septic patients [35]. Researchers in a controlled study reported that the prior statin therapy was associated with the decrease in inflammatory reactions in patients with severe sepsis [36].

Merx et al. [37] revealed that the average survival rate of septic animals that used statin was 4-fold higher than that of the control animals. Simvastatin reduced the recruitment and activation of inflammatory cells, thus protected against the LPS-induced ALI.

We found that simvastatin administration protected against septic ALI, as reflected by the marked amelioration of histological injury of lung tissues, and the decrease in inflammatory cell infiltration, and by an increase in GR, SOD, and GSH-Px antioxidant enzymes in LPS-treated rats. Reducing the levels of Galectin-3 may act as an endogenous compensatory anti-inflammatory mechanism in ALI.

In conclusion, our results might have important implications regarding the prior dosing of simvastatin administration as a potential protector against ALI.

## Abbreviations

ALI: acute lung injury
GR: glutathione reductase
GSH-Px: glutathione peroxidase
GSSG: glutathione
H&E: hematoxylin and eosin
HMG-CoA: 3-hydroxyl-3-methyl coenzyme A
IL: interleukin
LPS: lipopolysaccharides
ROS: reactive oxygen species
SD: standard deviation
SOD: superoxide dismutase
TBARS: thiobarbituric acid reactive substances
TLR4: toll-like receptor 4

## References

[1] Singer M., Deutschman C.S., Seymour C.W., Shankar-Hari M., Annane D., Bauer M.. (2016) The third international consensus definitions for sepsis and septic shock (Sepsis-3). J. Am. Med. Assoc. 315, 801–810 10.1001/jama.2016.0287 26903338 PMC4968574

[2] Xu C., Shi Q., Zhang L. and Zhao H. (2018) High molecular weight hyaluronan attenuates fine particulate matter-induced acute lung injury through inhibition of ROSASK1-p38/JNK-mediated epithelial apoptosis. Environ. Toxicol. Pharmacol. 59, 190–198 10.1016/j.etap.2018.03.020 29625389

[3] Han C.H., Guan Z.B., Zhang P.X., Fang H.L., Li L., Zhang H.M. (2018) Oxidative stress-induced necroptosis activation is involved in the pathogenesis of hyperoxic acute lung injury. Biochem. Biophys. Res. Commun. 495, 2178–2183 10.1016/j.bbrc.2017.12.100 29269294

[4] Hayes J.D. and McLellan L.I. (1999) Glutathione and glutathione-dependent enzymes represent a co-ordinately regulated defence against oxidative stress. Free Radic. Res. 31, 273–300 10.1080/10715769900300851 10517533

[5] Betteridge D.J. (2000) What is oxidative stress? Metabolism 49, 3–8 10.1016/S0026-0495(00)80077-3 10693912

[6] Wang P., Ye X.L., Liu R., Chen H.L., Liang X., Li W.L. (2013) Mechanism of acute lung injury due to phosgene exposition and its protection by caffeic acid phenethyl ester in the rat. Exp. Toxicol. Pathol. 65, 311 10.1016/j.etp.2011.10.001 22030112

[7] Cooper D., Iqbal A.J., Gittens B.R., Cervone C. and Perretti M. (2012) The effect of galectins on leukocyte trafficking in inflammation: sweet or sour? Ann. N. Y. Acad. Sci. 1253, 181–192 10.1111/j.1749-6632.2011.06291.x 22256855

[8] Li Y., Komai-Koma M., Gilchrist D.S., Hsu D.K., Liu F.T., Springall T. (2008) Galectin-3 is a negative regulator of lipopolysaccharide-mediated inflammation. J. Immunol. 181, 2781–2789 10.4049/jimmunol.181.4.2781 18684969

[9] Madrigal-Matute J., Lindholt J.S., Fernandez-Garcia C.E., Benito-Martin A., Burillo E., Zalba G. (2014) Galectin-3, a biomarker linking oxidative stress and inflammation with the clinical outcomes of patients with atherothrombosis. J. Am. Heart Assoc. 3, e000785, 10.1161/JAHA.114.000785 25095870 PMC4310363

[10] Almkvist J., Fäldt J., Dahlgren C. and Leffler H. Karlsson A. (2001) Lipopolysaccharide-induced gelatinase granule mobilization primes neutrophils for activation by Galectin-3 and Formylmethionyl-Leu-Phe. Infect. Immun. 69, 832–837 10.1128/IAI.69.2.832-837.2001 11159975 PMC97959

[11] Fermino M.L., Polli C.D., Toledo K.A., Liu F.T., Hsu D.K., Roque-Barreira M.C. (2011) LPS-induced galectin-3 oligomerization results in enhancement of neutrophil activation. PLoS ONE 6, e26004 10.1371/journal.pone.0026004 22031821 PMC3198732

[12] Karlsson A., Christenson K., Matlak M., Björstad A., Brown K.L., Telemo E. (2009) Galectin-3 functions as an opsonin and enhances the macrophage clearance of apoptotic neutrophils. Glycobiology 19, 16–20 10.1093/glycob/cwn104 18849325

[13] Weitz-Schmidt G., Welzenbach K., Brinkmann V, Kamata T, Kallen J, Bruns C (2001) Statins selectively inhibit leukocyte function antigen-1 by binding to a novel regulatory integrin site. Nat. Med. 7, 687–692 10.1038/89058 11385505

[14] Souza Neto JL, Araújo Filho I, Rego AC, Dominici VA, Azevedo IM, Egito ES (2006) Effects of simvastatin in abdominal sepsis in rats. Acta Cir. Bras. 21, 8–12 10.1590/S0102-86502006001000003 17293958

[15] Pierre-Paul D. and Gahtan V. (2003) Noncholesterol-lowering effects of statins. Vasc. Endovasc. Surg. 37, 301–313 10.1177/153857440303700501 14528375

[16] Fai Mok S.W., Thelen K.M., Riemer C., Bamme T., Gültner S., Lütjohann D. (2006) Simvastatin prolongs survival times in prion infections of the central nervous system. Biochem. Biophys. Res. Commun. 348, 697–702 10.1016/j.bbrc.2006.07.123 16890918

[17] Buege J.A. and Aust S.D. (1978) Microsomal lipid peroxidation methods. Methods Enzymol. 52, 302–310 10.1016/S0076-6879(78)52032-6 672633

[18] Babayigit H., Kucuk C., Sozuer E., Yazici C., Kose K. and Akgun H. (2005) Protective effect of beta-glucan on lung injury after cecal ligation and puncture in rats. Intensive Care Med. 31, 865–870 10.1007/s00134-005-2629-x 15818502

[19] Sakaguchi S., Kanda N., Hsu C.C. and Sakaguchi O. (1981) Lipid peroxide formation and membrane damage in endotoxin-poisoned mice. Microbiol. Immunol. 25, 229–244 10.1111/j.1348-0421.1981.tb00026.x 6973056

[20] Spapen H., Zhang H., Demanet C., Vleminckx W., Vincent J.L. and Huyghens L. (1998) Does N-acetyl-L-cysteine influence cytokine response during early human septic shock? Chest 113, 1616–1624 10.1378/chest.113.6.1616 9631802

[21] Flaherty J.T. and Weisfeldt M.L. (1988) Reperfusion injury. Free Radic. Biol. Med. 5, 409–419 10.1016/0891-5849(88)90115-3 3076884

[22] Yorulmaz H., Ozkok E., Ates G. and Tamer S. (2017) Investigation of the effectiveness of ghrelin treatment in lung tissue of rats with sepsis. Bratisl. Lek. Listy 118, 585–590 29198124 10.4149/BLL_2017_112

[23] Uysal M. and Karaman S. (2018) In vivo effects of intravenous lipid emulsion on lung tissue in an experimental model of acute malathion intoxication. Toxicol. Indus. Health 34, 110–118 10.1177/0748233717748080 29415640

[24] Napierala M., Merritt T.A., Mazela J., Jablecka K., Miechowicz I., Marszalek A. (2017) The effect of tobacco smoke on oxytocin concentrations and selected oxidative stress parameters in plasma during pregnancy and post-partum: an experimental model. Hum. Exp. Toxicol. 36, 135–145 10.1177/0960327116639363 27009111

[25] Cumaoğlu A., Ozansoy G., Irat A.M., Arıcıoğlu A., Karasu C. and Arı N. (2011) Effect of long term, non cholesterol lowering dose of fluvastatin treatment on oxidative stress in brain and peripheral tissues of streptozotocin-diabetic rats. Eur. J. Pharmacol. 654, 80–85 10.1016/j.ejphar.2010.11.035 21172345

[26] Stoll L.L., McCormick M.L., Denning G.M. and Weintraub N.L. (2004) Antioxidant effects of statins. Drugs Today 40, 975–989 10.1358/dot.2004.40.12.872573 15645009

[27] Kuwabara I. and Liu F.T. (1996) Galectin-3 promotes adhesion of human neutrophils to laminin. J. Immunol. 156, 3939–3944 8621934

[28] Sano H., Hsu D.K., Yu L., Apgar J.R., Kuwabara I., Yamanaka T. (2000) Human galectin-3 is a novel chemoattractant for monocytes and macrophages. J. Immunol. 165, 2156–2164 10.4049/jimmunol.165.4.2156 10925302

[29] Sato S., Ouellet N., Pelletier I., Simard M., Rancourt A. and Bergeron M.G. et al. (2002) Role of galectin-3 as an adhesion molecule for neutrophil extravasation during streptococcal pneumonia. J. Immunol. 168, 1813–1822 10.4049/jimmunol.168.4.1813 11823514

[30] Henderson N.C. and Sethi T. (2009) The regulation of inflammation by galectin. Immunol. Rev. 230, 160–171 10.1111/j.1600-065X.2009.00794.x 19594635

[31] Novak R., Dabelic S. and Dumic J. (2012) Galectin-1 and galectin-3 expression profiles in classically and alternatively activated human macrophages. Biochim. Biophys. Acta 1820, 1383–1390 10.1016/j.bbagen.2011.11.014 22155450

[32] Alves C.M., Silva D.A., Azzolini A.E., Marzocchi-Machado C.M., Carvalho J.V., Pajuaba A.C. (2010) Galectin-3 plays a modulatory role in the life span and activation of murine neutrophils during early Toxoplasma gondii infection. Immunobiology 215, 475–485 10.1016/j.imbio.2009.08.001 19720428

[33] Lee Y.J., Koh Y.S., Park H.E., Lee H.J., Hwang B.H., Kang M.K. (2013) Spatial and temporal expression, and statin responsiveness of galectin-1 and galectin-3 in murine atherosclerosis. Korean Circ. J. 43, 223–230 10.4070/kcj.2013.43.4.223 23682281 PMC3654109

[34] Chopra M., Reuben J.S. and Sharma A.C. (2009) Acute lung injury: apoptosis and signaling mechanisms. Exp. Biol. Med. 4, 361–371 10.3181/0811-MR-318 19176873

[35] Kruger P., Fitzsimmons K., Cook D., Jone M. and Nimmo G. (2006) Statin therapy is associated with fewer deaths in patients with bacteraemia. Intensive Care Med. 32, 75–79 10.1007/s00134-005-2859-y 16283159

[36] Liappis A.P., Kan V.L., Rochester C.G. and Simon G.L. (2001) The effect of statins on mortality in patients with bacteremia. Clin. Infect. Dis. 33, 1352–1357 10.1086/323334 11565076

[37] Merx M.W., Liehn E.A., Graf J., van de Sandt A., Schaltenbrand M., Schrader J. (2005) Statin treatment after onset of sepsis in a murine model improves survival. Circulation 5, 117–124 10.1161/CIRCULATIONAHA.104.502195 15998696

